# Occipital lobe infarction: a rare presentation of bilateral giant cavernous carotid aneurysms: a case report

**DOI:** 10.1186/s12886-018-0687-4

**Published:** 2018-02-02

**Authors:** Kavin Vanikieti, Anuchit Poonyathalang, Panitha Jindahra, Piyaphon Cheecharoen, Wimonwan Chokthaweesak

**Affiliations:** 1 Department of Ophthalmology, Faculty of Medicine Ramathibodi Hospital, Mahidol University, 270 Rama VI Road, Bangkok, 10400 Thailand; 2 Department of Medicine, Faculty of Medicine Ramathibodi Hospital, Mahidol University, 270 Rama VI Road, Bangkok, 10400 Thailand; 3 Department of Radiology, Faculty of Medicine Ramathibodi Hospital, Mahidol University, 270 Rama VI Road, Bangkok, 10400 Thailand

**Keywords:** Bilateral, Giant, Cavernous carotid aneurysms, Occipital lobe infarction

## Abstract

**Background:**

Cavernous carotid aneurysm (CCA) represents 2–9% of all intracranial aneurysms and 15% of internal carotid artery (ICA) aneurysms; additionally, giant aneurysms are those aneurysms that are > 25 mm in size. Bilateral CCAs account for 11–29% of patients and are commonly associated with structural weaknesses in the ICA wall, secondary to systemic hypertension. CCAs are considered benign lesions, given the low risk for developing major neurologic morbidities (i.e., subarachnoid hemorrhage, cerebral infarction, or carotid cavernous fistula). Moreover, concurrent presentation with posterior circulation cerebral infarction is even rarer, given different circulation territory from CCA. Here, we report on a patient with bilateral giant CCAs who presented with both typical and atypical symptoms.

**Case presentation:**

An 88-year-old hypertensive woman presented with acute vertical oblique binocular diplopia, followed by complete ptosis of the right eye. Ophthalmic examination showed dysfunction of the right third, fourth, and sixth cranial nerves. Further examination revealed hypesthesia of the areas supplied by the ophthalmic (V1) and maxillary (V2) branches of the right trigeminal nerve. Bilateral giant cavernous carotid aneurysms, with a concurrent subacute right occipital lobe infarction, were discovered on brain imaging and angiogram. Additionally, a prominent right posterior communicating artery (PCOM) was revealed. Seven months later, clinical improvement with stable radiographic findings was documented without any intervention.

**Conclusions:**

Dysfunction of the third, fourth, and sixth cranial nerves, and the ophthalmic (V_1_) and maxillary (V_2_) branches of the trigeminal nerves, should necessitate brain imaging, with special attention given to the cavernous sinus. Despite unilateral symptomatic presentation, bilateral lesions cannot be excluded solely on the basis of clinical findings. CCA should be included in the differential diagnosis of cavernous sinus lesions. Although rare, ipsilateral posterior circulation cerebral infarction (i.e., occipital lobe infarction) can occur in CCA patients, presumably as a result of distal embolization through an ipsilateral, prominent PCOM. Spontaneous clinical improvement with stable radiographic support may occur.

## Background

Cavernous carotid aneurysm (CCA) represents 2–9% of all intracranial aneurysms and 15% of internal carotid artery (ICA) aneurysms [1], primarily affecting women across various age groups [1, 2]. Its etiology can be infectious, traumatic, or idiopathic. In a previous study, 32.5% of all CCAs were classified as giant aneurysms [1], which are > 25 mm in size. Bilateral CCAs are present in 11–29% of patients and are commonly associated with structural weakness of the ICA wall, secondary to systemic hypertension [1–5].

Given the essential structures within and adjacent to the cavernous sinus, most patients with CCA often present with neuro-ophthalmic manifestations, such as ocular motor paresis, trigeminal sensory neuropathy, retro-orbital pain, facial pain, Horner’s pupil, compressive optic neuropathy, and amaurosis fugax [1–5]. However, CCAs rarely present with cerebral infarction [2, 5], and even more rarely present with posterior circulation cerebral infarction, given that this type of infarct arises from a different portion of the circulation system, relative to CCAs. Here, we report on a patient with bilateral giant CCAs who presented with both typical and atypical symptoms.

## Case presentation

An 88-year-old Thai woman experienced acute vertical-oblique binocular diplopia, 10 days prior to presentation at our clinic. Three days later, she developed complete ptosis of the right eye, which eliminated her diplopia and was accompanied by mild retro-orbital pain on the right side. She denied history of previous trauma or febrile illness and did not notice any fluctuations in her symptoms. Her past medical history was significant for moderately controlled hypertension.

**Fig. 1:**
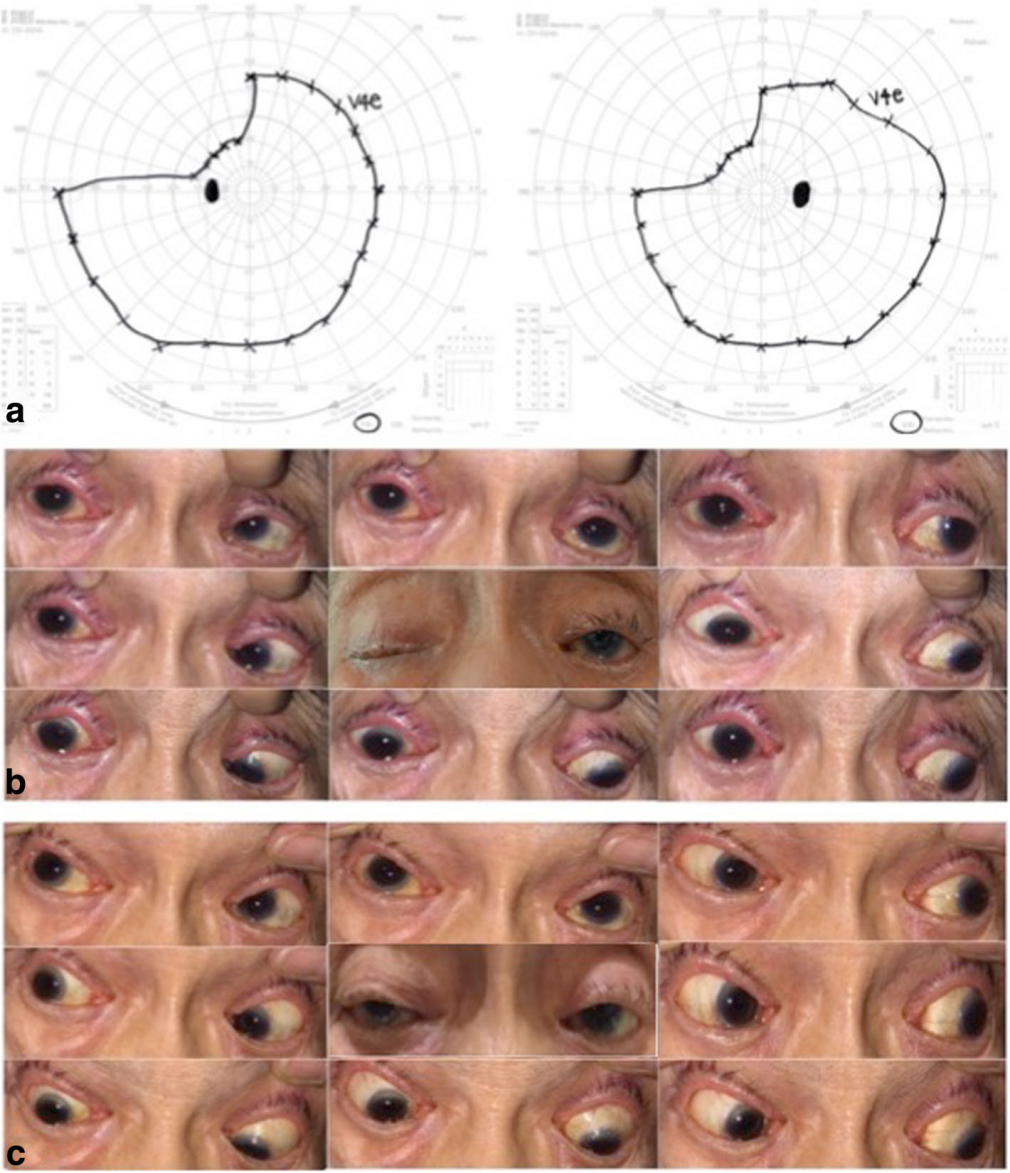
Visual field, external appearance, and ocular motility of the patient’s initial presentation. **a** Goldmann visual field testing reveals left superior homonymous quadrantanopia. **b** Complete ptosis of the right eye (center) and marked limitation of ocular motility of the right eye in all directions. **c** After 7 months of follow up, ptosis (center) and limitation of ocular motility of the right eye were less visible

Ophthalmic examination revealed a best corrected visual acuity of 20/25 in both eyes. Color vision test results were unremarkable. Goldmann visual field testing revealed left superior homonymous quadrantanopia (Fig. 1a). The right eye demonstrated complete ptosis and marked limitation of ocular motility in all directions (Fig. 1b). Furthermore, incyclotorsion of the right eye was not observed when infraduction was attempted. Right pupil measured 5 mm and responded sluggishly to light; in contrast, left pupil measured 3 mm and exhibited a brisk response to light. There was no relative afferent pupillary defect. Intraocular pressure, anterior segment, and dilated fundal examinations of both eyes were unremarkable. Neither proptosis, nor orbital bruit, was observed. Systemic neurological examination was significant for hypesthesia of the areas supplied by the ophthalmic (V_1_) and maxillary (V_2_) branches of the right trigeminal nerve.

Blood tests, including complete blood count (CBC), erythrocyte sedimentation rate (ESR), C-reactive protein (CRP) and coagulation, were normal.

**Fig. 2:**
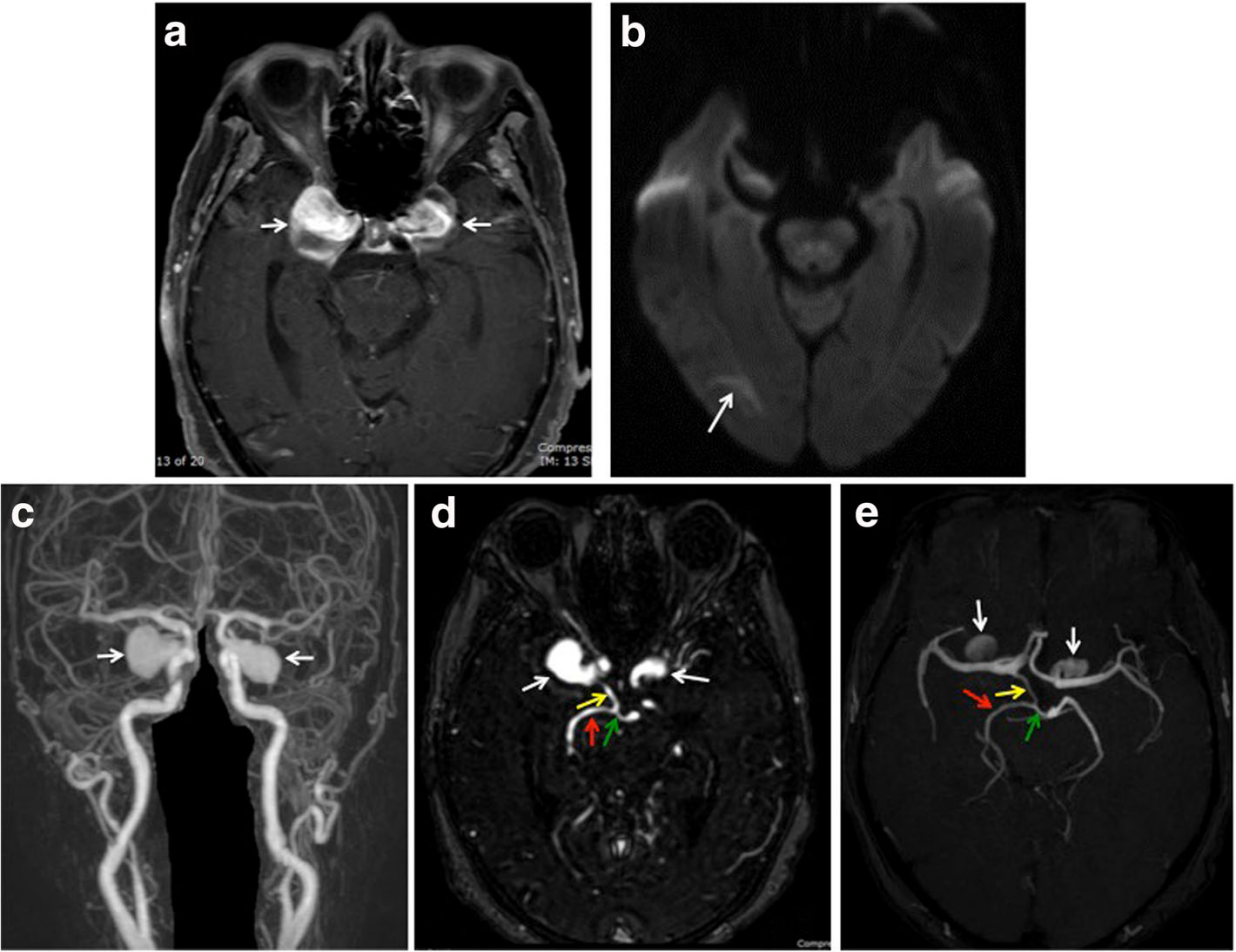
Magnetic resonance imaging (MRI) and magnetic resonance angiography (MRA) of the brain. **a** Contrasted MRI: partially thrombosed bilateral giant cavernous carotid aneurysms (CCAs, arrows). **b** Diffusion-weighted MRI: restricted diffusion of the right occipital lobe (arrow). **c–e** MRA: bilateral CCAs (white arrows) and dual vascular supply of right posterior cerebral artery (PCA, red arrows) from prominent posterior communicating artery (PCOM, yellow arrows) and P1 (green arrows). Left PCOM is absent

Magnetic resonance imaging (MRI) and magnetic resonance angiogram (MRA) of the brain revealed bilateral partially thrombosed giant CCAs, measuring 2.7 × 2.3 × 2.7 cm and 2.3 × 2.6 × 1.9 cm on the right and left sides, respectively (Fig. 2a–e). Additionally, dual vascular supply of the right posterior cerebral artery (PCA) was revealed; one supply arose from the P1 segment, while the other arose from the prominent right posterior communicating artery (PCOM) (Fig. 2d, e). No other concomitant aneurysms were detected outside of the cavernous carotid artery. No significant stenosis was noted in the vertebrobasilar arterial system. MRI of the brain also showed a subacute right occipital lobe infarction (Fig. 2b). Neither dilatation of superior ophthalmic veins, nor congestion of orbital contents, was observed.

In order to exclude a cardioembolic source, electrocardiogram and echocardiography were performed; these revealed normal results.

Diagnosis consisted of bilateral giant CCAs with concurrent subacute right occipital lobe infarction. Endovascular intervention was not performed, given the advanced age of the patient. Thus, daily aspirin was conservatively initiated to prevent further thromboembolic cerebral infarction.

Seven months later, the patient reported significant improvement of her symptoms. Retro-orbital pain on the right side was less prominent. Ptosis and limited ocular motility of the right eye were less visible (Fig. 1c). Both pupils were symmetric and responded well to light. Furthermore, equal sensation was reported on both sides in the areas supplied by the V_1_ and V_2_ branches of the trigeminal nerves. Compared with the size and extent of thrombosis on initial imaging findings, follow-up MRI and MRA of the brain revealed stable bilateral giant CCAs.

## Discussion

Our patient presented with concurrent typical and atypical symptoms. Similar to our current case, one previous report on 206 CCAs showed that diplopia was the most common presenting symptom (65%), followed by retro-orbital pain (34%). Moreover, the most common finding at presentation was a cavernous sinus syndrome that involved a combination of the third, fourth, and sixth cranial nerves (18% of 206 CCAs) [2].

The lack of neuro-ophthalmic symptoms and findings related to the left CCA was not surprising, as asymptomatic CCAs are found in 18% of CCA cases [2]. Notably, the right CCA was larger than the left CCA, which may explain the disparity in symptomatic presentation. Compared with the left CCA, we suspect that the right CCA had become large enough to exhibit mass effects on structures within the cavernous sinus.

Although they are rare, other major neurologic morbidities related to CCA are: 1) cerebral infarction from spontaneous occlusion of the ipsilateral ICA or distal embolization of thrombus within the aneurysm, and 2) carotid cavernous fistula (CCF) and subarachnoid hemorrhage (SAH) as sequelae of a ruptured CCA [5].

In our patient, subacute right occipital lobe infarction was demonstrated on initial MRI of the brain; this corresponded to left superior homonymous quadrantanopia in Goldmann visual field testing. A previous study revealed that only 1% of CCAs develop ipsilateral cerebral hemisphere infarction, and that all patients with CCA-related cerebral infarction exhibited pre-existing systemic coagulopathy; further, the infarction involved only territory within the anterior circulatory system [5]. However, we suspect that the subacute right occipital lobe infarction in our patient was related to right CCA, based on the following reasons:

1. Distal embolization of thrombus within the right CCA, via a prominent right PCOM
2. Absence of significant stenosis and other concomitant aneurysms in the vertebrobasilar arterial system.
3. Absence of a cardioembolic source, because of normal results on electrocardiogram and echocardiography.

Without any intervention, improvement of diplopia and concurrent reduction of pain have both been reported. One study showed that, if left untreated, either diplopia or pain improved in 56% of CCAs. Based on that report, selection bias—related to severity of symptoms—has impeded comparison of outcomes and complications between untreated and treated patients [2]. Our patient showed spontaneous marked clinical improvement with stable radiographic support and conservative aspirin therapy.

## Conclusions

Dysfunction of the third, fourth, and sixth cranial nerves, and the ophthalmic (V_1_) and maxillary (V_2_) branches of the trigeminal nerves, should necessitate brain imaging, with special attention given to the cavernous sinus. Despite unilateral symptomatic presentation, bilateral lesions cannot be excluded solely on the basis of clinical findings. CCA should be included in the differential diagnosis of cavernous sinus lesions. Although rare, ipsilateral posterior circulation cerebral infarction (i.e., occipital lobe infarction) can occur in CCA patients, presumably as a result of distal embolization through an ipsilateral, prominent PCOM. Spontaneous clinical improvement with stable radiographic support may occur.

## Abbreviations

CBC: Complete blood count
CCA: Cavernous carotid aneurysm
CCF: Cavernous-carotid fistula
CRP: C-reactive protein
ESR: Erythrocyte sedimentation rate
ICA: Internal carotid artery
MRA: Magnetic resonance angiography
MRI: Magnetic resonance imaging
PCA: Posterior cerebral artery
PCOM: Posterior communicating artery
SAH: Subarachnoid hemorrhage
V_1_: Ophthalmic
V_2_: Maxillary

## References

[1] Rosi Junior J, Welling LC, Yeng LT, Caldas JG, Schafranski M, Teixeira MJ, Figueiredo EG (2014). Cavernous carotid artery aneurysms: epidemiology, natural history, diagnostic and treatment. An experience of a single institution. Clin Neurol Neurosurg.

[2] Stiebel-Kalish H, Kalish Y, Bar-On RH, Setton A, Niimi Y, Berenstein A, Kupersmith MJ (2005). Presentation, natural history, and management of carotid cavernous aneurysms. Neurosurgery.

[3] Ambekar S, Madhugiri V, Sharma M, Cuellar H, Nanda A (2014). Evolution of management strategies for cavernous carotid aneurysms: a review. World Neurosurg.

[4] Goldenberg-Cohen N, Curry C, Miller NR, Tamargo RJ, Murphy KP (2004). Long term visual and neurological prognosis in patients with treated and untreated cavernous sinus aneurysms. J Neurol Neurosurg Psychiatry.

[5] Kupersmith MJ, Stiebel-Kalish H, Huna-Baron R, Setton A, Niimi Y, Langer D, Berenstein A (2002). Cavernous carotid aneurysms rarely cause subarachnoid hemorrhage or major neurologic morbidity. J Stroke Cerebrovasc Dis.

